# Editorial: Updates in urogynecological surgery

**DOI:** 10.3389/fsurg.2024.1363416

**Published:** 2024-02-05

**Authors:** Stefano Cianci

**Affiliations:** Department of Human Pathology of Adult and Childhood “G. Barresi” Unit of Gynecology and Obstetrics, University of Messina, Messina, Italy

**Keywords:** urogynecology, gynecologic surgery, pelvic organ prolapse, urinary incontinence, quality of life

**Editorial on the Research Topic** Updates in urogynecological surgery

Urogynecology stands at the forefront of women's health, addressing the intricate web of challenges associated with pelvic floor disorders. The prevalence of conditions such as urinary incontinence and pelvic prolapse underscores the profound impact on the well-being and quality of life for countless individuals (1). Within the realm of urogynecology, surgical interventions play a pivotal role in restoring normalcy to the lives of those affected by urinary incontinence and pelvic prolapse. Various surgical techniques have emerged as cornerstones of treatment, each with its unique merits and considerations (2).

Surgical approaches for urinary incontinence, such as sling procedures and retropubic suspensions, have demonstrated efficacy in restoring bladder control. The evolution of these techniques, marked by refinements in methodology and enhanced patient outcomes, reflects the dynamism of urogynecological surgery. Similarly, the management of pelvic prolapse has witnessed significant strides. From traditional approaches like hysterectomy to more contemporary methods like mesh augmentation, surgeons navigate a spectrum of options. Recent studies illuminate the nuanced outcomes associated with different procedures, offering valuable insights into tailoring treatments to individual patient needs (3, 4).

As we delve into the intricacies of urogynecological surgery, it becomes evident that a personalized and evidence-based approach is paramount. The convergence of surgical expertise, technological innovation, and patient-centered care defines the current landscape and paves the way for future advancements in the field.

Beyond the operating room, understanding the origins of pelvic dysfunction is pivotal in advancing urogynecological care. Pelvic health is a tapestry woven with genetic predispositions, hormonal intricacies, and lifestyle factors. Unraveling this complexity is essential for tailoring interventions and addressing the root causes of pelvic issues.

Recent research endeavors have illuminated the genetic underpinnings of pelvic floor disorders, providing insights into hereditary influences that may predispose individuals to these conditions. Concurrently, hormonal fluctuations throughout a woman's life, from puberty to menopause, exert profound effects on pelvic structures. Exploring the interplay between hormones and pelvic health opens avenues for targeted therapeutic interventions (5).

Lifestyle factors, encompassing everything from physical activity to diet and childbirth practices, contribute significantly to pelvic dysfunction. A comprehensive understanding of these influences empowers healthcare providers to offer holistic guidance and preventive measures.

The etiological landscape of pelvic dysfunction is dynamic and evolving. As researchers delve deeper into the molecular and environmental factors, the potential for more precise diagnostics and personalized treatment strategies becomes increasingly promising.

The heartbeat of urogynecology resonates with innovation, with each stride bringing forth novel technologies and approaches. In recent years, scientific breakthroughs have transformed diagnostics, surgical precision, and postoperative care, heralding a new era in pelvic health.

Cutting-edge technologies in imaging, such as three-dimensional ultrasound and magnetic resonance imaging, provide unprecedented clarity in assessing pelvic anatomy. These advancements not only enhance preoperative planning but also contribute to more accurate diagnoses (6).

Surgical innovations, including minimally invasive techniques and robotic-assisted procedures, have revolutionized the landscape of urogynecological interventions. The quest for less invasive yet highly effective approaches reflect a commitment to optimizing patient outcomes and expediting recovery (7).

Diagnostic tools, such as urodynamics and biomarker assessments, continue to evolve, offering clinicians a more nuanced understanding of pelvic function and dysfunction. The integration of these tools into routine practice refines diagnostic accuracy and informs tailored treatment plans.

As we stand on the cusp of a new era in urogynecology, the synergy between technological innovation and clinical acumen holds the promise of elevating patient care to unprecedented heights. Continued collaboration between researchers, practitioners, and technologists ensures that the momentum of innovation propels the field toward ever-greater advancements.

At the heart of urogynecology lies a profound commitment to improving patient outcomes and enhancing the quality of life for individuals grappling with pelvic floor disorders. The success of surgical interventions and innovative treatments is measured not only in clinical metrics but, more importantly, in the tangible impact on the lives of those we serve (8).

In this Research Topic, we embark on a journey through the latest advancements in urogynecology, exploring surgical interventions, unraveling the cure of pelvic dysfunction, and shedding light on groundbreaking innovations that are reshaping the landscape of women's healthcare.

It was a pleasure for me to serve as Guest Editor of the Research Topic of *Frontiers in Surgery* entitled *Updates in Urogynecological Surgery*. This Research Topic was conceptualized with the aim to give an overview on latest innovation in the field of urogynecology. A series of articles produced by proven experts in the field of gynecology has been presented regarding different aspects of urogynecology. I believe that the subjects explored in this Research Topic will be of interest to a wide audi­ence, from basic academic researchers to clini­cians in gynecology, oncologists, and surgeons.

The Research Topic starts with the first article by. Ronsini et al. entitled *Laparoscopic uterosacral ligament suspension: a systematic review and meta-analysis of safety and durability*. The authors focused their research on a specific procedure made for pelvic prolapse reporting interesting data through a meta-analysis. The second article by Panico et al. entitled *The first 60 cases of robotic sacrocolpopexy with the novel HUGO RAS system: feasibility, setting and perioperative outcomes* reported the preliminary results on surgical application of a new robotic platform for sacrocolpopexy procedure.

The third article by Song et al. entitled *Case report: The intrauterine suture surgery—an original method in the treatment of old uterine false passage* reports a technique of uterine suture.

The fourth article by Devassy et al. entitled *Modified Oxford technique of colpopexy for the treatment of uterine and vaginal vault prolapse: a retrospective pilot cohort study* reported some data related to a surgical technique applied for vaginal prolapse.

The fifth article by Drusany Starič et al. entitled *Delayed surgical management of rectovaginal fistula: a case report highlighting challenges and lessons learned* reports on a case of surgical repair of a recto-vaginal fistula.

The last article by Malanowska-Jarema et al. entitled *A comparative study in learning curves of laparoscopic lateral suspension vs. laparoscopic sacrocolpopexy: preliminary results* reported interesting data about the learning curve of a surgical procedure for pelvic organ prolapse.

I would like to thank all authors for these fine and interesting articles which makes a significant contribution on scientific panorama.

As we reflect on the current landscape of urogynecology, the path forward is both promising and laden with challenges. The relentless pursuit of knowledge and innovation opens avenues for transformative advancements, but it also unveils gaps in our understanding that demand meticulous exploration.

Future research endeavors in urogynecology are poised to unravel the intricacies of individualized medicine. Genetic profiling, biomarker identification, and the integration of artificial intelligence into diagnostics herald a future where treatment plans are tailored with unprecedented precision.

In conclusion, urogynecology stands as a beacon of progress in women's health, tackling the intricate challenges woven into the fabric of pelvic floor disorders. From the surgical suite to the realms of genetics and innovation, the field is marked by continuous evolution and a steadfast commitment to patient-centered care.

As we celebrate the achievements and advancements, we acknowledge that the journey is far from complete. Urogynecology is a dynamic field, propelled by the collective efforts of researchers, clinicians, and patients. The chapters yet to be written hold the promise of deeper understanding, refined interventions, and, most importantly, an improved quality of life for those entrusted to our care.
